# Determinants of tetanus, pneumococcal and influenza vaccination in the elderly: a representative cross-sectional study on knowledge, attitude and practice (KAP)

**DOI:** 10.1186/s12889-016-2784-8

**Published:** 2016-02-04

**Authors:** Carolina J. Klett-Tammen, Gérard Krause, Linda Seefeld, Jördis J. Ott

**Affiliations:** 1 Department of Epidemiology, Helmholtz Centre for Infection Research, Inhoffenstrasse 7, 38124 Braunschweig, Germany; 2 Hannover Medical School, Carl-Neuberg-Str. 1, 30625 Hannover, Germany; 3 Unit of Basic medical issues; preventive and medical activities in health education, Federal Centre for Health Education (BZgA), Maarweg 149-161, 50825 Köln, Germany

**Keywords:** Pneumococcal vaccine, Influenza vaccine, Tetanus vaccine, Aged, Health knowledge, Attitude, Practice

## Abstract

**Background:**

Severity and incidence of vaccine-preventable infections with influenza viruses, s*. pneumoniae* and *c. tetani* increase with age. Furthermore, vaccine coverage in the elderly is often insufficient. The aim of this study is to identify socio-economic and knowledge-, attitude- and practice- (KAP)-related determinants of vaccination against influenza, pneumococcal disease and tetanus in the older German population.

**Methods:**

We analysed data from a German nationally representative questionnaire-based KAP-survey on infection prevention and hygiene behavior in the elderly (*n* = 1223). We used logistic regressions to assess impacts of socio-demographic- and KAP-related variables on vaccine uptake in general and on tetanus-, influenza- and pneumococcal vaccination. To generate KAP-scores, we applied factor analyses and analysed scores as predictors of specific vaccinations.

**Results:**

A low rated personal health status was associated with a higher uptake of influenza vaccine whereas place of residence within Germany strongly impacted on pneumococcal vaccination. For tetanus and influenza vaccination, the strongest single vaccination predictor was attitude-related, i.e., the perceived importance of the vaccine (OR = 18.1, 95 % CI = 4.5–71.8; OR = 23.0, 95 % CI = 14.9–35.3, respectively). Pneumococcal vaccination was mostly knowledge-associated, i.e., knowing the recommendation predicted uptake (OR = 17.1, 95 % CI = 9.5–30.7). Regarding the generated KAP-scores, the practice-score reflecting vaccine related behavior such as having a vaccination record, was predictive for all vaccines considered. The knowledge-score was associated with influenza (OR = 1.3, 95 % CI = 1.0–1.6) and pneumococcal vaccination (OR = 1.2, 95 % CI = 1.0–1.5). Uniquely for influenza vaccination, the attitude-score was linked to vaccine uptake (OR = 1.1, 95 % CI = 1.0–1.1).

**Conclusions:**

Our results indicate that predictors of vaccination uptake in the elderly strongly depend on vaccine type and that scores of KAP are useful and valid to condense information from numerous individual KAP-variables. While awareness for vaccinations against influenza and tetanus is fairly high already it might have to be increased for vaccinations against pneumocoocal infections.

**Electronic supplementary material:**

The online version of this article (doi:10.1186/s12889-016-2784-8) contains supplementary material, which is available to authorized users.

## Background

Older age is strongly associated with disease severity and case fatality resulting from *Streptococcus pneumoniae* (*s. pneumoniae*) [1], influenza virus infection [2], and infection with *Clostridium tetani*. [3] Severe disease outcomes from *S. pneumoniae* include invasive pneumococcal diseases (IPD) [4], as sepsis, pneumonia [5], and meningitis [6], whereas influenza virus infection is characterized by high fever, aching muscles, headache, cough, sore throat and rhinitis. Tetanus mostly presents as a spastic disease, followed by spastic paralysis and death. In Germany, reported incidence of IPD and influenza infection in 2014 was 1.35/100.000 and 5.92/100.000, respectively in the age group 60 years and older. There was no reported case of tetanus [7]. Since there is no nationwide mandatory reporting for IPD in Germany, reported incidence is likely to be substantially underestimated [8, 9]. Vaccines against all three diseases are available and German vaccine recommendations for adults aged ≥60 years include the 10 year interval tetanus and diphtheria vaccination, the annual influenza vaccination and a singular pneumococcal vaccination, using the 23-valent PPV dose, while PCV is also licensed [10]. The self-reported vaccination rate in the German 60–79 years old population varies by vaccine type and ranges from 31 % for pneumococcal, to 66 % for influenza and 70 % for tetanus vaccination [11]. While a national campaign promotes influenza vaccination [12], no comparable strategies are in place for pneumococcal or tetanus vaccination, focusing on the elderly.

Health-related behavior is affected by different aspects of knowledge, attitude and practices (KAP) [13]. Reviewing the existing evidence from 69 international publications on determinants of vaccine-usage in the elderly, we found the following to be most frequently significantly associated with vaccine uptake: socio-demographic determinants such as living arrangement [14], low awareness of the vaccine recommendation [15, 16], attitudes like the perceived low severity of the corresponding disease [17, 18], and practices including previous uptake of vaccinations [19]. Little is known about determinants of uptake of vaccinations in the older German population and how they vary by vaccine type. Furthermore, most studies investigated associations of single variables particularly with influenza vaccine uptake, while less attention is paid to officially recommended tetanus and pneumococcal vaccination.

Using data of a representative national survey on infection prevention, the objective of this study was to assess associations between socio-demographic- and KAP-factors and vaccine uptake in order to identify determinants of vaccine uptake in the elderly. Using multivariate and factor analyses, knowledge-, attitude- and practice-related predictors of tetanus, influenza and pneumococcal vaccination were analysed. Given the high information load created when assessing KAP-variables on an individual level, we tested and applied statistical methods to create scores of variables with statistically significant associations.

## Methods

### Study design and data management

We analysed data from a German nationally representative cross-sectional survey on infection prevention, initiated by the Federal Centre for Health Education (BZgA) in 2012. Reference population for representativeness was the German national Census [20] including an error tolerance of +/−3 %. Inclusion criteria for the study were: ability to understand and speak German, age between 16 and 85 years, living in a private household in Germany. The sample was drawn via the ADM-telephone-master-sample for both, household phones and mobile phones. In each of the reached households, the household member whose birthday was last, was chosen as participant [21]. Computer-Assisted Telephone Interviews were conducted using a questionnaire that contains 101 questions on vaccination-related KAP leading to 112 variables. The full list of variables of the data set with original coding and recoding is available from Additional file 1. For all variables in the data set, missing values were less than 5 % and were at random and excluded using list wise deletion. Further methodological details of the overall survey are described elsewhere [21].

### Data analysis

Outcomes we considered were self-reported influenza-, pneumococcal-, tetanus- and any vaccination in the last 5 years. Independent variables were socio-demographic, health-, knowledge-, attitude- and practice-related variables. We conducted descriptive analysis and bivariate analyses including chi^2^-tests to assess associations between socio-demographic and vaccination-related KAP-variables, and self-reported vaccine uptake (crude risk ratios for dichotomous variables). Potential effect modification and/or confounding by age, sex, migration-status, place of residence, and education was addressed by Mantel-Haenszel tests. To generate odds ratios (OR) for effects of KAP-related variables on the self-reported uptake of influenza-, pneumococcal-, tetanus- and any vaccination, we applied adjusted logistic regression, and used hierarchical backward elimination of non-significant (α > 0.05) variables and interactions (if change of estimate for residual variables = <10 %) for best model fit [22, 23], separately for each vaccine and their combination. We assessed validity of final models by likelihood-ratio test and Nagelkerke’s r-square.

In a second step, we composed scores of KAP. Given the large sample size (>1000 participants) and the high participant-variable ratio (1:18 at minimum) [24], explanatory principal axis factor analyses with oblique promax-rotation and Kaiser-normalization was used. We selected and aggregated most relevant individual predictors within each score according to scree-plot and the Kaiser-criterion, as available from Additional file 2. Factors with loadings <0.4 were excluded and confirmatory factor analyses were conducted. We applied a Cronbach’s alpha threshold of 0.70 to assure internal validity.

After dichotomization of each score by median (higher versus lower vaccination-related KAP), we conducted multiple logistic score regression analyses for associations between scores and influenza-, pneumococcal-, tetanus- and any vaccination, respectively. We compared results from score regression models to those from regression models conducted using individual variables.

Statistical analyses were done using Stata IC 12; for factor analyses we used SPSS 20.

## Results

### Descriptive results (bivariate analysis)

The sample consisted of 4483 German adults (16 to 85 years) of which 1223 individuals were in the upper age class (60 to 85 years, mean: 69.2 years, SD: 0.6) and were included in the analyses. The majority of participants was female (57.8 %) and had an educational level of secondary education or higher (66.7 %). 12.5 % had a migration background (Table 1).

**Table 1:** Baseline characteristics, study population > =60 years., *n* = 1 223

Variable	N (%)
Age-groups	
60–65	448 (36.6)
66–70	246 (20.1)
71–75	308 (25.2)
76–80	151 (12.4)
81–85	70 (5.7)
Sex	
Female	707 (57.8)
Male	516 (42.2)
Migration background (defined by country of birth and nationality of participant and parents)	
No	1070 (87.5)
Yes	153 (12.5)
Education	
No graduation/Certificate of Secondary Education	395 (32.3)
General Certificate of Secondary Education	363 (29.7)
Polytechnic degree or higher	453 (37.0)
Missing/don’t know	12 (1.0)
Place of residence	
Western part of Germany	890 (72.8)
Eastern part of Germany (incl. Berlin)	328 (26.8)
Missing/don’t know	5 (0.4)
Subjective health status	
(very) good	606 (49.5)
Average	440 (36.0)
(very) bad	176 (14.4)
Missing/don’t know	1 (0.1)
Chronic condition	
No	726 (59.4)
Yes	495 (40.5)
Missing/don’t know	2 (0.2)
Vaccine-Recommendation by a physician	
No	875 (71.5)
Yes	345 (28.2)
Missing/don’t know	3 (0.3)

Half of the respondents rated their health status as “good” or “very good”, although 40.5 % indicated having a chronic condition of any kind. Vaccination, independent of the type of vaccine, had been recommended by a physician to 28.2 % of the participants. 72.0 % of the participants indicated they were vaccinated during the last 5 years whereas uptake varied by vaccine type ranging from 11.5 % for pneumococcal vaccination to 50 % for annual influenza and 56.3 % for tetanus vaccination (Table 2).

**Table 2:** Uptake of vaccinations, study population > =60 years, *n* = 1 223

Variable	N (%)
Any vaccination in last 5 years	881 (72.0)
Missings/ don’t know	5 (0.4)
Tetanus-vaccination in last 5 years	688 (56.3)
Missings/don’t know	8 (0.7)
Pneumococcal-vaccination in last 5 years	140 (11.5)
Missings/don’t know	22 (1.8)
Influenza-vaccination in last 5 years	629 (51.4)
Missings/don’t know	1 (0.1)
Influenza-vaccination ever	817 (66.8)
Missings/don’t know	3 (0.3)
Influenza-vaccination annually	586 (47.9)
Missings/don’t know	3 (0.3)

50.2 % of respondents rated pneumococcal vaccination as important. Pneumococcal, tetanus and influenza vaccine recommendations were known to 28.2, 72.9, and 78.9 % of participants, respectively. Almost 2/3 of the participants had a positive general attitude towards vaccination and 55 % trusted the official vaccine recommendations. More than 90 % perceived physicians to be an appropriate source of information regarding vaccinations (Additional file 3).

### Individual predictors of vaccination (multivariate logistic regression)

- Any vaccination
  Results from the multivariate analysis revealed that practice-related variables such as the intention to get vaccinated against influenza (OR = 6.9, CI = 4.0–12.0) and the possession of a vaccination record (OR = 4.5, CI = 2.7–7.4) was strongly associated with vaccine uptake in general (Table 3).

**Table 3:** Determinants of uptake of vaccinations in general and for specific vaccines, multivariate logistic regression^a^

	Any vaccination^b^		Tetanus^c^		Influenza^d^		Pneumococcal^e^	
Nagelkerkes r^2^	39.0 %		24.8 %		55.7 %		49.3 %	
	OR (95 % CI)	*p*-value	OR (95 % CI)	*p*-value	OR (95 % CI)	*p*-value	OR (95 % CI)	*p*-value
Variable								
Knowledge-related variables								
Recommendation diphtheria- vaccination, spontaneous	3.7 (2.0–6.9)	<0.001						
Recommendation pneumococcal-vaccination, spontaneous							4.5 (2.2–9.0)	<0.001
Recommendation influenza vaccination if asked	2.0 (1.3–2.9)	<0.01						
Recommendation influenza-vaccination-time interval upon request					4.2 (2.4–7.4)	<0.001		
Recommendation pneumococcal vaccination if asked							17.1 (9.5–30.7)	<0.001
Attitude-related variables								
Attitude towards vaccinations -neutral	2.4 (1.3–4.2)	<0.01			2.1 (0.8–5.6)	0.1		
-(rather) positive	2.7 (1.5–4.7)	<0.01			3.4 (1.3–8.9)	0.01		
Vaccination due to physician recommendation					3.1 (2.1–4.7)	<0.001		
Vaccination due to familiy member recommendation					19.8 (4.8–82.1)	<0.001		
Vaccination due to travel					2.5 (1.6–3.7)	<0.001		
Vaccination to protect others					3.6 (1.7–7.5)	<0.01		
Vaccination due to media information			3.9 (2.9–5.3)	<0.001				
Vaccination due to occupational exposure			3.3 (2.2–5.1)	<0.001				
Barrier to influenza vaccine uptake: fear of needles			0.3 (0.1–0.8)	0.02				
Perceived importance of pneumococcal-vaccination							5.7 (2.8–11.4)	<0.001
Perceived importance of Influenza-vaccination	1.8 (1.2–2.9)	<0.01			23.0 (14.9–35.3)	<0.001		
Perceived importance of Tetanus-vaccination			18.1 (4.5–71.8)	<0.001				
Practice-related variables								
Possession of vaccination record	4.5 (2.7–7.4)	<0.001						
Refusal of some vaccination	0.6 (0.4–0.9)	0.02						
Intention to get influenza-vaccination	6.9 (4.0–12.0)	<0.001						
Receipt of annual influenza vaccination	1.9 (1.2–2.8)	<0.01						
Receipt of Tetanus-vaccination in previous 5 years					3.0 (2.0–4.5)	<0.001	2.1 (1.2–3.9)	0.02
Influenza-vaccination in previous 5 years			3.9 (3.0–5.2)	<0.001			7.4 (3.6–15.1)	<0.001
Pneumococcal-vaccination in previous 5 years					3.8 (1.8–8.3)	0.001		
Interaction: sex- perceived infromation level	women: 0.4 (0.2–0.7)	<0.01	men: 2.3 (1.4–3.6)	<0.001				

^a^only variables with a significant association in the final models are shown ^b^adjusted for sex, perceived information level and recommendation by physician, ^c^adjusted for sex and perceived information level, ^d^adjusted for recommendation by physician and subjective rated health status, ^e^adjusted for place of residence
- Tetanus vaccination
  Participants who considered it as important to be vaccinated against tetanus were 18 times more likely to be vaccinated. Being vaccinated due to media information (OR = 3.9, 95 % CI = 2.9–5.3) and occupational exposure (OR = 3.3, 95 % CI = 2.2–5.1) also increased the chance of having a tetanus vaccination. The perceived level of information available to an individual was a sex-dependent predictor for the uptake of the tetanus vaccination with men being more likely to be vaccinated against tetanus, if feeling well informed. Most predictors for the tetanus vaccination were attitude-related while none was knowledge-related (Table 3).
- Influenza vaccination
  Similar to the tetanus vaccination, the most important predictor for being vaccinated against influenza was the perceived importance of the influenza vaccination, with a 23 times higher likelihood for being vaccinated. For influenza vaccine specifically, uptake was mostly associated with attitude-related variables whereas socio-demographic factors showed no impact. Regarding the practice of vaccine uptake by an individual, the recommendation by a physician significantly increased the likelihood of being vaccinated (OR = 1.7, CI = 1.1–2.7). A low rated own health status was positively correlated with influenza vaccine uptake compared to a good subjective health status (OR = 2.9, CI = 1.1–3.3) (Table 3).
- Pneumococcal vaccination
  Knowledge-related variables such as being aware of the vaccine recommendation were strongly associated with pneumococcal vaccine uptake. Similar to influenza and tetanus vaccination but to a lesser degree, the high perceived importance of the vaccine was related to a higher chance to be vaccinated (six times as high for pneumococcal vaccine, 23 and 14 times as high for influenza and tetanus vaccine, respectively).

Among the socio-demographic variables, place of residence (Eastern versus Western Germany) influenced uptake of the pneumococcal vaccination: Participants living in the Eastern part of Germany were 80 % more likely (OR = 1.8, CI = 1.1–3.1) to be vaccinated against pneumococci (Table 3).

### Scores as predictors for vaccination (multivariate score analysis)

Factor analyses resulted in three scores which consisted of two (knowledge-score) or five factors (attitude- and practice-score). All Cronbach’s alphas were at above 0.70. Details on score generation are available from Additional file 4 and Additional file 5.

The multivariate score analysis aggregated individual KAP-variables. The result for “any vaccination” revealed that respondents with a higher practice-score, i.e., those who indicated to perform more vaccination-related practices like having a vaccination record or consulting vaccination advice, had an almost four times higher likelihood for receipt of a vaccination compared to those with a lower practice-score. The knowledge- and attitude-score were not significantly associated with uptake of “any vaccination” (Table 4). Similar results were observed for tetanus vaccine uptake. For influenza vaccine, all three scores determined the chance of being vaccinated: participants with a higher knowledge-score were 30 % more likely to get vaccinated compared to those with a lower score. A higher practice-score indicated a 40 % higher likelihood to receive pneumococcal vaccine (Table 4).

**Table 4:** Synopsis of final models of logistic regressions: Scores as predictors of uptake of vaccinations ^a^

Scores	Any vaccination		Tetanus		Influenza^b^		Pneumococcal	
Nagel-kerkes r^2^	64.7 %		40.6 %		57.8 %		20.6 %	
	OR (95 % CI)	*p*-value	OR (95 % CI)	*p*-value	OR (95 % CI)	*p*-value	OR (95 % CI)	*p*-value
Knowledge- Score	Not significant		Not significant		1.3 (1.0–1.6)	0.02	1.2 (1.0–1.5)	<0.05
Attitude- Score	Not significant		Not significant		1.1 (1.0–1.1)	<0.001	Not significant	
Practice- Score	3.9 (3.3–4.7)	<0.001	2.1 (1.9–2.2)	<0.001	2.5 (2.2–2.8)	<0.001	1.4 (1.3–1.5)	<0.001

^a^adjusted for age and recommendation received by physician, if not stated otherwise, ^b^adjusted for age and subjective rated health status

### Comparison of individual variables and score analysis for predicting vaccine uptake

Regarding “any vaccination”, the comparison of the variables-based and the score-based analysis revealed the same result, i.e., that the general vaccination practice is the most important predictor for vaccine uptake.

Results for score-based and variable-based analysis differed when looking at specific vaccines. For influenza, attitude served as a predictor on both, the variable- and score-level, while the scores “knowledge” and “practice” additionally determined influenza vaccine receipt. Tetanus vaccination was mostly related to attitudes as reflected by the individual attitude-related determinants. Controversially, in the score-based analysis the strongest association with the uptake of the tetanus vaccination was shown for the practice-score. The comparison of the individual variable- and the score-analysis for pneumococcal vaccination revealed that knowledge and practice are predictors on both, the individual variable and score level (Table 5).

**Table 5:** Comparison of predictors: variables vs. multivariate score-analyses

	Any vaccination	Tetanus vaccination	Influenza vaccination	Pneumococcal vaccination
Variables				
Most predictors related to	Practice	Attitude	Attitude	Knowledge, Practice
Variable with highest impact related to	Practice	Attitude	Attitude	Knowledge
Scores	Practice	Practice	Knowledge, Attitude, Practice	Knowledge, Practice

## Discussion

This study is the first one focusing on KAP related to three different vaccinations, recommended for the elderly in Germany. Most vaccine-related surveys concentrate on parents of children [25] or on adolescents [26]. Elderly are less frequently the target group of investigations on vaccine-KAP. However, given their vulnerability for severe infectious disease outcomes [1–3] and different exposures to infectious agents (e.g., through their living environment), hygiene behavior and infection prevention including vaccination is of relevance in this age-stratum. We found great variations in predictors of vaccination in the elderly, depending on vaccine type considered, which may allow conclusions on vaccine type-specific public health action needed. Overall, practices such as possessing a vaccination record were predictive for uptake of all vaccines analysed, while the attitude towards vaccination was particularly associated with the influenza vaccine usage. For pneumococcal vaccination, knowledge was positively associated with vaccine utilization. Furthermore, by aggregating individual KAP-variables into three scores, we showed that for any, influenza, and pneumococcal vaccination the generated scores equally reflect the information from the individual variables. For tetanus vaccination, however individual variables of attitude were most important but on a score level, the practice score was the only significant predictor. This could imply that general vaccine attitudes do not influence the uptake of tetanus vaccination, but only those attitudes related to this specific vaccine. On the other hand, general vaccination-related practices as possessing a vaccination record were associated with higher tetanus vaccination uptake. No individual knowledge-related variable was related to the uptake of the tetanus vaccination, which differs from pneumococcal vaccination. As we also found that most elderly in Germany (almost 80 % of the participants) were well informed about the existing recommendation, promotion activities regarding tetanus immunization may rather focus on individual attitudes than on information provision in general. Exclusively for the influenza vaccine, the attitude-score showed a significant influence on uptake, corresponding with the strong association with attitude-related single variables. This may reflect the controversial public discussions regarding the usefulness of this vaccine. As all scores were associated with uptake of the influenza vaccination, all areas of KAP might be equally important targets for promoting influenza vaccination.

Different from influenza vaccination, where attitude-related variables served as best predictors for vaccine uptake, in case of the pneumococcal vaccination, individual attitude-related variables did not have a notable impact on the pneumococcal vaccination and less than 30 % of the participants knew about the recommendation. This contrasts with the almost 80 % who were aware of the influenza vaccination recommendation. Based on these findings, the main reason for the low uptake of the pneumococcal immunization among the elderly in Germany might be the lack of awareness and not a general refusal of this vaccine. Also in other risk groups knowledge about pneumococcal disease and the vaccination was found to be low [27]. The association of knowledge with uptake of the pneumococcal vaccination has been shown in previous studies. Schneeberg et al. [28] illustrated that the strongest predictor for pneumococcal vaccination was the recommendation by a health care worker (HCW) and the knowledge of the vaccine [28]. Similarly, Johnson et al. [29] found that 79–85 % would get vaccinated with pneumococcal vaccine if a HCW would offer and recommend it. In another study, 91.3 % of the participants who were not vaccinated against pneumococci stated this was because they did not know about the recommendation or their physician did not recommend it [30].

The impact of knowledge and awareness on pneumococcal vaccine uptake shown from our study could warrant nationwide campaigns for pneumococcal vaccine in Germany, or implementation of public health measures as done for influenza vaccine. The potential need for vaccine information and promotion activities is additionally reflected by the relatively low vaccine coverage shown in our study, particularly for pneumococcal vaccine. The coverage in our study was also lower compared to those reported from previous investigations in Germany. For example, Poethko-Müller et al. [11] reported coverage of about 70 % for tetanus vaccine, about 66 % for influenza vaccine and about 31 % for pneumococcal vaccine in individuals aged 60–79 years [11] versus 56.3 % for tetanus vaccine, 50 % for influenza vaccine and 11.5 % for pneumococcal vaccine in individuals aged 60–85 years in our study. These differences might result from the fact that in our study it was only asked for vaccinations received during the last 5 years. As tetanus vaccination is generally given in a 10 year interval and pneumococcal vaccinations just once, the answers from our study may not reflect “coverage” as such.

The main strength of our study is the representativeness of the sample, providing information on vaccine-related KAP among the older population of Germany, for the first time. Generating all three KAP-scores based on factor analyses has, to our knowledge, not been applied in the area of vaccination-related KAP-surveys in the elderly but may assist in drawing conclusions from numerous KAP-specific variables without losing important information. Similar results obtained from the variable- versus score-specific analyses furthermore suggest that the scores adequately reflect individual variables in each of the three KAP categories. Up to our knowledge, only one published study used scores based on factor analysis to investigate KAP related to vaccination against Human Papilloma Virus [31]. Despite the similar approach used in this investigation, attitude- and belief-related determinants but no variables on knowledge and practice were included. There was no direct comparison made between score- and variable-based results, which is, in our opinion, crucial to determine the validity of results. Having said this, we assessed each multivariate model by calculating Nagelkerke’s r^2^ and found a high degree of explained variance from 20.6 to 64.7 %, meaning that a great amount of the outcome can be explained by the determinants in our models.

As a limiting factor and inherent in the design of a cross-sectional study, we were not able to assess the timing of the vaccinations and its direct predictors. Thus, it is possible that immunization status itself has influenced knowledge or attitudes, which both might have been different before getting vaccinated. Since public awareness activities mainly target influenza vaccination in Germany, this possibly also affected knowledge and attitudes towards this vaccination but also the self-reported vaccine-uptake for this vaccination. Although the attitude-related questions targeted some personal beliefs, for example the trust in official vaccine recommendations, there was no question precisely assessing risk perceptions such as vaccine safety concerns or perceived seriousness of infection in detail. This information may have provided additional information for interpreting our results and should be included in follow-up investigations of this kind.

Despite the availability of safe vaccines, the proportion of vaccinated elderly is low and beyond recommended coverage in Germany. This is furthermore remarkable with regard to demographic changes and the impact vaccine-preventable diseases have on the health outcomes of the elderly. In our study population vaccination was recommended to only 29 % of the participants by their physician. Thus, the result that vaccine uptake in the elderly is strongly influenced by practices like getting a vaccination consultation, is of high importance to health care providers. The significance of the physician- patient- interaction based on knowledge and attitudes of the health care professionals for the uptake of vaccination in this age-strata, has also been shown in another study [32].

## Conclusions

We analysed associations of KAP with the uptake of specific vaccinations in the elderly in Germany, using logistic regressions and score building within a representative sample. Our findings suggest that there are different patterns for the different vaccine types, leading to the necessity of specific public health actions for each vaccine. While for the pneumococcal vaccine the knowledge about the recommendation needs to be addressed, for the influenza vaccine mainly the attitudes towards the vaccination are of interest and for the tetanus vaccination general vaccination-related practices, as possessing a vaccination record, should be tackled. In general, many elderly have not received advice regarding recommended vaccinations by their physician. This results in a need for integrating health care workers in the research of determinants of vaccine uptake in the elderly in Germany and in the design of related promotion activities, taking into consideration implications of varying vaccine performance, i.e., efficacy of each of the vaccines in general and in the elderly [33–38].

### Ethics approval and consent to participate

All procedures performed in this study involving human participants were in accordance with the institutional and national research committee and with the 1964 Helsinki declaration and its later amendments or comparable ethical standards. The original collection of the data was performed on behalf of the Federal Centre for health education (BZgA), in accordance to the ESOMAR-Code. As data used for these analyses did not include individual personal data or personal identifiers, ethical approval was not required.

### Availability of data and materials

Data and questionnaires used were available from the Federal Centre for Health Education (BZgA) (Head 1985–2015, Professor Pott. Dataset and questionnaires were sent on behalf of Professor Pott). In timely alignment with this study, the data set has been made available as a public use file in November 2015 on GESIS - Leibniz-Institut für Sozialwissenschaften: Datenbestandskatalog.

## Additional files

Additional file 1: **List of all variables in the data set used in analyses, original coding and recoding.** Description: lists all variables that were used in these analyses. Original coding and recoding are provided. Descriptions of the variables are given. (PDF 648 kb)

Additional file 2: **Variables included in the factor analyses.** Description: lists all variables that were included in the factor analyses in the areas of knowledge, attitude and practices. (PDF 880 kb)

Additional file 3: **Further description of KAP in the study population.** Description: provides details on vaccination-related knowledge, attitude and practices in the study population (n = 1 223). (PDF 404 kb)

Additional file 4: **Details on results of factor analyses.** Description: describes in detail the generated factors which are the basis for the scores used in further analyses including KMO, Bartlett, Eigenvalue, factor loading, included variables, Cronbach’s alpha, mean and standard deviation. (PDF 441 kb)

Additional file 5: **Characteristics of scores.** Description: shows the characteristics of the generated scores in terms of mean, standard deviation, minimum, maximum, median and numbers and percentages regarding the dichotomized scores. (PDF 169 kb)
