# Supplementary material for: Determinants of tetanus, pneumococcal and influenza vaccination in the elderly: a representative cross-sectional study on knowledge, attitude and practice (KAP)
Source: BMC Public Health. 2016 Feb 4;16:121. doi: 10.1186/s12889-016-2784-8 (PMC4743086; doi:10.1186/s12889-016-2784-8)
Supplement: Supplementary file 1 — List of all variables in the data set used in analyses, original coding and recoding. Description: lists all variables that were used in these analyses. Original coding and recoding are provided. Descriptions of the variables are given. (PDF 648 kb) [file 12889_2016_2784_MOESM1_ESM.pdf]

Additional file 1: List of all variables in the data set used in analyses, original coding and recoding

Name	Description	Original coding	Recoding	Comment
**Socio-demographic**				
age	age in years	year of birth	2012-year of birth	
	age in five-year increments		in five-year increments	
sex	sex	1=male<br>2=female	0=female<br>1=male	
Migration background	Any migration background of the participant or his/her parents	Each question: all countries mentioned were coded as one variable, with codings<br>0=no<br>1=yes	0=no migration background<br>1=migration background	Describes whether a participant was born in Germany, has the German nationality, whether his/her parents are of German nationality, and whether parents were born in Germany. If at least one of these criteria was not applicable, the participant was considered as having migration background (coded with 1).
Education	School education	1=no graduation<br>2=Certificate of Secondary Education (9 yrs schooling)<br>3=General Certificate of Secondary Education (10 yrs schooling)<br>4=Polytechnic Secondary School<br>5=Polytechnic degree<br>6=general qualification for university entrance<br>7=Advanced technical college or University<br>8=other<br>9=Don´t know/No answer	1 and 2→1= no graduation/Certificate of Secondary Education<br>3 and 4→2= General Certificate of Secondary Education<br>5, 6 and 7→3= polytechnic degree or higher<br><br>8 and 9→ missing	
Place of residence	Describes whether the participant lives in the eastern (newly-formed German states) or western part of Germany.	Community-indicator, continuous	0=Western part of Germany<br>1=Eastern part of Germany (incl. Berlin)	Federal state (first two digits) was extracted and allocated to east and west of Germany.
**Health related**				
Subjective health status	Rating of own health	1=very good<br>2=good<br>3=average<br>4=bad<br>5=very bad<br>6=Don´t know/ No	1=(very) good<br>2= average<br>3=(very) bad	

		answer	
Chronic condition	Information on chronic condition of the participant (not further specified)	1=yes<br>2=no<br>3=Don´t know/ No answer	0=No<br>1=Yes
Recommendation by a physician	A physician has recommended to get vaccinated during the previous 12 months	1=Yes<br>2=No<br>3=Don´t know/ No answer	0=No<br>1=Yes
**Knowledge**			
Perceived information level	How the participant perceives his/her information level regarding vaccinations in adulthood	1= Very good<br>2=Good<br>3=Less good<br>4=Bad<br>5=Don´t know/ No answer	1 and 2→0=(very) good<br>3 and 4→1=(rather) bad
Recommendations by STIKO	Knowledge on who is in charge of official vaccine recommendations	1= Federal Ministry of Health and its technical authorities<br>2=Pharmaceutical industry<br>3= health insurance companies<br>4= committee of independent experts<br>5=Don´t know/ No answer	0=Wrong<br>4→1=Correct
Awareness of official influenza-campaign by BZgA/RKI	Awareness of the official influenza vaccination campaign run by the BZgA/RKI: Ever seen/read posters, advertisement in magazine or information brochure	1=Yes, read or seen<br>2=No<br>3=Don´t know/ No answer	0=No<br>1=Yes
Awareness of brochure by BZgA/RKI	Ever seen/read the brochure of the BZgA/RKI about immunization protection for the whole family	1=Yes, read or seen<br>2=No<br>3=Don´t know/ No answer	0=No<br>1=Yes
Awareness of official measles-campaign by BZgA	Awareness of the official Measles-Mumps-Rubella vaccination campaign by the BZgA: Ever seen/read brochures/posters	1=Yes, read or seen<br>2=No<br>3=Don´t know/ No answer	0=No<br>1=Yes
Recommendation for pneumococcal vaccination known, if asked	Interviewer mentioned the official recommendation for pneumococcal vaccination for persons > 59 years and asked whether the participant was aware of this recommendation	1=Yes<br>2=No<br>3=Don´t know/ No answer	0=No<br>1=Yes
Recommendation for influenza vaccination	Interviewer mentioned the official	1=Yes<br>2=No	0=No<br>1=Yes

known, if asked	recommendation for influenza vaccination for persons > 59 years and asked afterwards whether the participant was aware of this recommendation	3=Don´t know/ No answer		
Recommendation Tetanus-vaccination known, spontaneous	Does the participant know spontaneously that the tetanus vaccination is recommended in adulthood	0=Not mentioned<br>1=mentioned	0=No<br>1=Yes	It was asked which vaccinations have to be repeated in adulthood, every possible answer was coded in a separate variable
Recommendation Diphtheria-vaccination known, spontaneous	Does the participant know spontaneously that the diphtheria vaccination is recommended in adulthood	0=Not mentioned<br>1=mentioned	0=No<br>1=Yes	It was asked which vaccinations have to be repeated in adulthood, every possible answer was coded in a separate variable
Recommendation Influenza-vaccination known, spontaneous	Does the participant know spontaneously that the influenza vaccination is recommended in adulthood	0=Not mentioned<br>1=mentioned	0=No<br>1=Yes	It was asked which vaccinations have to be repeated in adulthood, every possible answer was coded in a separate variable
Recommendation Pneumococcal-vaccination known, spontaneous	Does the participant know spontaneously that the pneumococcal vaccination is recommended in adulthood	0=Not mentioned<br>1=mentioned	0=No<br>1=Yes	It was asked which vaccinations have to be repeated in adulthood, every possible answer was coded in a separate variable
Recommended Tetanus-vaccination time interval	In which time interval the tetanus vaccination has to be repeated according to participants opinion	1=once<br>2=every ten years<br>3=every three to five years<br>4=every year<br>5=Don´t know/No answer	0=wrong (incl. not mentioned)<br>1=right (every ten years)	If a vaccination in the question above was mentioned, it was asked, in which time interval it has to be repeated
Recommended Diphtheria-vaccination time interval	In which time interval the diphtheria vaccination has to be repeated according to participants opinion	1=once<br>2=every ten years<br>3=every three to five years<br>4=every year<br>5=Don´t know/No answer	0=wrong (incl. not mentioned)<br>1=right (every ten years)	If a vaccination in the question above was mentioned, it was asked, in which time interval it has to be repeated
Recommended Influenza-vaccination-time interval	In which time interval the influenza vaccination has to be repeated according to participants opinion	1=once<br>2=every ten years<br>3=every three to five years<br>4=every year<br>5=Don´t know/No answer	0=wrong (incl. not mentioned)<br>1=right (every year)	If a vaccination in the question above was mentioned, it was asked, in which time interval it has to be repeated.
**Attitude**				
Attitude towards vaccinations	Attitude towards vaccinations in general	1=negative<br>2=rather negative<br>3=neutral<br>4=rather positive<br>5=positive<br>6=Don´t know/ No answer	1=(rather) negative<br>2=neutral<br>3=(rather) positive	
Trust in official vaccination	Trust in persons who are responsible for	1=Yes<br>2=No	0=No<br>1=Yes	

recommendations	official vaccination recommendations: Are their decisions evidence-based?	3=Don´t know/ No answer		
Appropriate source of information<br><br>-Internet<br>-Physician<br>-Physician´s assistant<br>-Brochure<br>-Newspaper/magazine<br>-TV<br>-Informations in schools<br>-Friends<br>-Family<br>-Federal and national state authorities<br>-Pharmaceutical enterprises<br>-Health office<br>-Health insurance company	Appropriate source of information regarding vaccinations and hygiene	1=Yes<br>2=No<br>3=Don´t know/ No answer	0=No<br>1=Yes	Participant indicated yes/no according to source.
Vaccination due to<br><br>- Vaccination due to media information<br>- Vaccination to protect others<br>- Vaccination due to occupational exposure<br>- Vaccination due to travel<br>- Vaccination due to Recommendation	*Motivation for a vaccination that was given in the previous five years*	*1=Yes*<br>*2=No*<br>*3=Don´t know/No answer*	*0=No (incl. no vaccination)*<br>*1=Yes*	*Participant indicated yes/no according to source.*
→*Recommendation by*<br><br>- Vaccination due to physician recommendation<br>- Vaccination due to recommendation by Health insurance company<br>- Vaccination due to familiy member recommendation<br>- Vaccination due to recommendation by friends<br>- Vaccination due to recommendation by others	*Source of recommendation of vaccination that led to vaccination*	*0= No*<br>*1=Yes*	*0=No*<br>*1=Yes*	*If answer to Motivation recommendation was yes, it was asked by whom. Multiple answers were possible. Every possible answer was coded in a separate variabl.e*
Barriers to vaccine	*Reasons for not*	*1=Yes*	*0=No (incl.no*	*If Participant refused a*

uptake	*getting vaccinated*	*2=No*<br>*3=Don´t know/ No answer*	*vaccination refused)*<br>*1=Yes*	*vaccination in previous years, it was asked why. Participant indicated yes/no according to reason. Every possible answer was coded in a separate variable.*
-Barrier *to vaccine uptake*: disease perceived as harmless				
-Barrier *to vaccine uptake:* fear of side effects				
-Barrier *to vaccine uptake:* fear of needles				
- *Barrier to vaccine uptake*: forgotten appointment				
- *Barrier to vaccine uptake:* belief that vaccine offers no protection				
-Barrier *to vaccine uptake:* discouraged by physician opinion				
-Barrier *to vaccine uptake:* discouraged by family/friends opinion				
-Barrier *to vaccine uptake:* critical reports in media				
-Barrier *to vaccine uptake:* too much effort				
-Barrier *to vaccine uptake:* general objection	general objection against vaccination			

Barriers to influenza vaccine uptake	*Reasons for not getting vaccinated against influenza*	*1=Yes*<br>*2=No*<br>*3=Don´t know/ No answer*	*0=No (incl.no vaccination refused)*<br>*1=Yes*	*If Participant did not receive influenza vaccination annually, reasons were asked. Participant indicated yes/no according to source. Every possible answer was coded in a separate variable.*
- Barrier to influenza vaccine uptake: disease harmless	Disease is perceived harmless			
- Barrier to influenza vaccine uptake : fear of side effects	Fear of side effects			
- Barrier to influenza vaccine uptake: fear of needles	Fear of needles			
- Barrier to influenza vaccine uptake: forgotten appointment				
- Barrier to influenza vaccine uptakea: belief that vaccine offers no	vaccination does not provide protection			

protection				
- Barrier to influenza vaccine uptake: contraindication	Participant cannot be vaccinated against influenza due to (possible) contraindication			
- Barrier to influenza vaccine uptake: discouraged by physician opinion				
- Barrier to influenza vaccine uptake: received no information	Participant did not know about the recommendation			
- Barrier to influenza vaccine uptake: does not belong to target group	Participant does not think he is in the target group			
Rating importance of particular vaccination	*How important is it to be vaccinated against:*	*1=Very important<br>2=important<br>3=Not important<br>4=Don´t know/ No answer*	*0=Not important/don´t know<br>1=(very) important*	*Perceived importance of vaccination was asked according to vaccination.*
Perceived importance of tetanus-vaccination				
Perceived importance of diphtheria-vaccination				
Perceived importance of influenza-vaccination				
Perceived importance of pneumococcal-vaccination				
Health consciousness	How much attention is paid to own health	1=very strong<br>2=strong<br>3=mediocre<br>4=less strong<br>5=not at all<br>6=Don´t know/ No answer	1= (very) strong<br>2=mediocre<br>3=less strong/not at all	
**Practice**				
Consulted web page „Impfen-Info.de“		1=Yes<br>2=No<br>3=Don´t know/ No answer	0=No<br>1=Yes	
Possession of a vaccination record		1=Yes<br>2=No<br>3=Don´t know/ No answer	0=No<br>1=Yes	
Consulting advice on vaccination	Consulted advice on vaccination in previous two years	1=Yes<br>2=No<br>3=Don´t know/ No answer	0=No<br>1=Yes	
Consulting advice on vaccination from	*Who gave advice?*	*1=Yes<br>2=No<br>3=Don´t know/ No answer*	*0=No<br>1=Yes*	*If Participant consulted advice for vaccination during the previous two years, it was asked where.*
Physician				
Pharmacist				
physician´s assistant				
Travel medicine clinic				
Health office				

Health insurance company				
Refusal of some vaccinations	Participant refused at least one vaccination in previous years	1=Yes<br>2=No<br>3=Don´t know/ No answer	0=No<br>1=Yes	
Any vaccination during previous five years	Participant received any vaccination during previous five years	1=Yes<br>2=No<br>3=Don´t know/ No answer	0=No<br>1=Yes	
Specific vaccinations during previous five years<br><br>Tetanus-vaccination during previous five years<br>Diphtheria-vaccination during previous five years<br>Influenza-vaccination during previous five years<br>Pneumococcal-vaccination during previous five years		*1=Yes*<br>*2=No*<br>*3=Don´t know/ No answer*	*0=No (incl. no vaccination at all)*<br>*1=Yes*	
Ever received influenza vaccination	Participant has ever been vaccinated against influenza		0=No<br>1=Yes	Variable was calculated:<br>0= Influenza vaccination during previous five years=0 & Influenza more than five years ago (not used in analysis)=0//<br>1=Influenza vaccination during previous five years or more than five years ago=1
Receipt of annual influenza vaccination	Participant gets vaccinated against influenza annually	1=Yes<br>2=No<br>3=Don´t know/ No answer	0=No<br>1=Yes	
Intention to get influenza-vaccination	Intention to get vaccinated against influenza next season	1=Yes<br>2=No<br>3=Don´t know/ No answer	0=No<br>1=Yes	Only asked if participant ≥60 yrs. or if health care worker
