# Supplementary material for: Determinants of tetanus, pneumococcal and influenza vaccination in the elderly: a representative cross-sectional study on knowledge, attitude and practice (KAP)
Source: BMC Public Health. 2016 Feb 4;16:121. doi: 10.1186/s12889-016-2784-8 (PMC4743086; doi:10.1186/s12889-016-2784-8)
Supplement: Supplementary file 2 — Variables included in the factor analyses. Description: lists all variables that were included in the factor analyses in the areas of knowledge, attitude and practices. (PDF 880 kb) [file 12889_2016_2784_MOESM2_ESM.pdf]

Additional file 2- Variables included in the factor analyses

Knowledge	Attitude	Practice
Recommendation Influenza-vaccination-time interval if asked	Health consciousness	Any vaccination during previous five years
Activator for Influenza-vaccination	Perceived importance of Diphteria-vaccination	Tetanus-vaccination during previous five years
Recommendations by STIKO	Perceived importance of Pertussis-vaccination	Diphteria-vaccination during previous five years
Recommendation Pertussis-vaccination if asked	Perceived importance of Polio-vaccination	Pneumococcal-vaccination during previous five years
Recommendation Influenza-vaccination if asked	Perceived importance of Hepatitis B-vaccination	Pertussis-vaccination during previous five years
Recommendation Pneumococcal-vaccination if asked	Perceived importance of Measles-vaccination	Polio-vaccination during previous five years
Recommendation Amarillic typhus-vaccination, spontaneous	Perceived importance of Mumps-vaccination	Hepatitis B-vaccination during previous five years
Recommendation Diphteria-vaccination, spontaneous	Perceived importance of Rubella-vaccination	Measles-vaccination during previous five years
Recommendation Hepatitis-A/B-vaccination, spontaneous	Perceived importance of Varicella-vaccination	Mumps-vaccination during previous five years
Recommendation Influenza-vaccination, spontaneous	Perceived importance of Meningococci-vaccination	Rubella-vaccination during previous five years
Recommendation Pertussis-vaccination, spontaneous	Perceived importance of Influenza-vaccination	Varicella-vaccination during previous five years
Recommendation Pneumococcal-vaccinations, spontaneous	Perceived importance of Pneumococcal-vaccination	Meningococcal-vaccination during previous five years
Recommendation Tetanus-vaccination, spontaneous	Vaccination due to physician recommendation	Influenza-vaccination during previous five years
Recommendation Influenza-vaccination-time interval	Vaccination due to recommendation byHealth insurance company	Influenza-vaccination ever
Recommended Diphteria-vaccination time interval	Vaccination due to family member recommendation	Influenza-vaccination annually
Recommended Tetanus-vaccination time interval	Vaccination due to friends recommendation	intention to receive influenza-vaccination
Perceived information level	Vaccination due to recommendationby others	Visited website
Awareness of official influenza-campaign by BZgA/RKI	Vaccination due to media information	How many times visited website
Awareness of brochure by BZgA/RKI	Vaccination to protect others	Possession vaccination record
Awareness of official measles-campaign by BZgA	Vaccination due to occupational exposure	Knowing where vaccination record is
	Vaccination due to travel	Vaccination consultation
	Barrier to influenza vaccine uptake: disease perceived as harmless	Vaccination consultation by Physician
	Barrier to influenza vaccine uptake: fear of side effects	Vaccination consultation by Pharmacist
	Barrier to influenza vaccine uptake: fear of needles	Vaccination consultation by physician´s assistant
	Barrier to influenza vaccine uptake: forgot appointment	Vaccination consultation by Travel medicine clinic
	Barrier to influenza vaccine uptake: belief that vaccine offers no protection	Vaccination consultation by Health office
	Barrier to influenza vaccine uptake: contraindication	Vaccination consultation by Health insurance company
	Barrier to influenza vaccine uptake: discouraged by physician opinion	Refused vaccination
	Barrier to influenza vaccine uptake: received no information	
	Barrier to influenza vaccine uptake: does not belong to target group	
	Preference of activator influenza vaccine	

	Trust in official recommendations	
	Barrier to vaccine uptake: disease perceived as harmless	
	Barrier to vaccine uptake: fear of side effects	
	Barrier to vaccine uptake: fear of needles	
	Barrier to vaccine uptake: forgot appointment	
	Barrier to vaccine uptake: belief that vaccine offers no protection	
	Barrier to vaccine uptake: discouraged by physician opinion	
	Barrier to vaccine uptake: discouraged by family/friends opinion	
	Barrier to vaccine uptake: critical reports in media	
	Barrier to vaccine uptake: too much effort	
	Barrier to vaccine uptake: general objection	
	Interest in further information	
	Interest in further information about side effects	
	Interest in further information about mode of functioning	
	Interest in further information about period of effect	
	Interest in further information about recommendations	
	Interest in further information about new developments	
	Interest in further information about assumption of costs	
	Internet appropriate	
	Physician appropriate	
	Physician´s assistant appropriate	
	Brochure appropriate	
	Newspaper/magazine appropriate	
	TV appropriate	
	Informations in schools appropriate	
	Friends appropriate	
	Family appropriate	
	Federal and national state authorities appropriate	
	Pharmaceutical enterprises appropriate	
	Health office appropriate	
	Health insurance company appropriate	
	Social networks appropriate	
	Attitude towards vaccinations	
