# Supplementary material for: Determinants of tetanus, pneumococcal and influenza vaccination in the elderly: a representative cross-sectional study on knowledge, attitude and practice (KAP)
Source: BMC Public Health. 2016 Feb 4;16:121. doi: 10.1186/s12889-016-2784-8 (PMC4743086; doi:10.1186/s12889-016-2784-8)
Supplement: Supplementary file 3 — Further description of KAP in the study population. Description: provides details on vaccination-related knowledge, attitude and practices in the study population (n = 1 223). (PDF 404 kb) [file 12889_2016_2784_MOESM3_ESM.pdf]

Additional file 3- Further description of KAP in the study population (n=1 223)

**Variable**	**N (%)**
Knowledge	
Subjectively informed	
(Rather) good	738 (64.0)
(Rather) bad	426 (34.8)
Missings/Don´t know	14 (1.1)
Official recommendations by STIKO	
Wrong/Don´t know	1026 (83.9)
Correct	197 (16.1)
Recommendation pneumococcal vaccination if asked	
No	878 (71.8)
Yes	345 (28.2)
Recommendation influenza vaccination if asked	
No	251 (20.5)
Yes	965 (78.9)
Missings	7 (0.6)
Recommendation Tetanus-vaccination, spontaneous	
No	332 (27.2)
Yes	891 (72.9)
Recommendation Diphtheria-vaccination, spontaneous	
No	1043 (85.3)
Yes	180 (14.7)
Recommendation Pneumococcal-vaccination, spontaneous	
No	1154 (94.4)
Yes	69 (5.6)
Recommendation Influenza- vaccination, spontaneous	
No	258 (21.1)
Yes	965 (78.9)
Attitude	
Attitude towards vaccinations	
(Rather) negative	118 (9.7)
Neutral	336 (27.5)
(Rather) positive	766 (62.6)
Missings/Don´t know	3 (0.3)
Trust in official recommendations	
No	488 (40.0)
Yes	677 (55.4)
Missings/Don´t know	58 (4.7)
Health conscious	
(very) strong	798 (65.3)
Mediocre	375 (30.7)
less strong/not at all	49 (4.0)
Missings/Don´t know	1 (0.1)
Appropriate source of information	
Physician	1114 (91.1)
Missings/Don´t know	11 (0.9)
Health insurance company	904 (73.9)
Missings/Don´t know	15 (1.2)
TV	833 (68.1)
Missings/Don´t know	15 (1.2)
Newspaper/magazine	760 (62.1)
Missings/Don´t know	9 (0.8)
Family	718 (58.7)
Missings/Don´t know	12 (1.0)
Brochure	715 (58.5)
Missings/Don´t know	17 (1.4)
Health office	715 (58.5)
Missings/Don´t know	21 (1.7)
Physician Assistant	636 (52.0)
Missings/Don´t know	16 (1.3)
Friends	631 (51.6)

Missings/Don´t know	11 (0.9)
Federal and Federal State Authorities	611 (50.0)
Missings/Don´t know	31 (2.5)
Informations in schools	443 (36.2)
Missings/Don´t know	29 (2.4)
Internet	418 (34.2)
Missings/Don´t know	24 (2.0)
Pharmaceutical enterprises	145 (11.9)
Missings/Don´t know	18 (1.5)
Social networks	91 (7.4)
Missings/Don´t know	34 (2.8)
Motivation	
Recommendation	507 (41.5)
Missings/Don´t know	5 (0.4)
Recommendation by physician	461 (36.9)
Missings/Don´t know	0 (0)
TV	448 (36.6)
Missings/Don´t know	24 (2.0)
Vacation	389 (31.8)
Missings/Don´t know	4 (0.3)
Work	233 (19.5)
Missings/Don´t know	8 (0.7)
Protection of others	128 (10.5)
Missings/Don´t know	17 (1.4)
Recommendation by family	30 (2.5)
Recommendation by others	28 (2.3)
Recommendation by friends	12 (1.0)
Recommendation by Health insurance company	4 (0.3)
Barriers	
Disease harmless	116 (9.5)
Missings/Don´t know	5 (0.4)
Side effects	95 (7.8)
Missings/Don´t know	1 (0.1)
No protection	93 (7.6)
Missings/Don´t know	7 (0.6)
Critical reports in media	58 (4.7)
Missings/Don´t know	4 (0.3)
Forgot appointment	57 (4.7)
Missings/Don´t know	1 (0.1)
General objection	41 (3.4)
Missings/Don´t know	1 (0.1)
Discouraged by physician opinion	39 (3.2)
Missings/Don´t know	2 (0.2)
Too much effort	25 (2.0)
Missings/Don´t know	2 (0.2)
Discouraged by family/friends opinion	23 (1.9)
Missings/Don´t know	1 (0.1)
Needles	6 (0.5)
Missings/Don´t know	1 (0.1)
Rating importance by vaccine type	
Tetanus (very) important	1178 (96.3)
Diphtheria (very) important	840 (68.7)
Influenza (very) important	704 (57.6)
Pneumococcal (very) important	614 (50.2)
Practice	
Possession vaccination record	1048 (85.7)
Missings/Don´t know	10 (0.8)
Vaccination consultation	271 (22.2)
Missings/Don´t know	3 (0.3)
Refused vaccination	254 (20.8)
Missings/Don´t know	22 (1.8)
Any vaccination during previous five years	881 (72.0)
Missings/Don´t know	5 (0.4)

Tetanus-vaccination during previous five years	688 (56.3)
Missings/Don´t know	8 (0.7)
Diphtheria-vaccination during previous five years	139 (11.4)
Missings/Don´t know	23 (1.9)
Pneumococcal-vaccination during previous five years	140 (11.5)
Missings/Don´t know	22 (1.8)
Influenza-vaccination during previous five years	629 (51.4)
Missings/Don´t know	1 (0.1)
Influenza-vaccination ever	817 (66.8)
Missings/Don´t know	3 (0.3)
Influenza-vaccination annually	586 (47.9)
Missings/Don´t know	3 (0.3)
Vaccination consultation by	
Physician	266 (21.8)
Physician´s Assistant	22 (1.8)
Travel medicine clinic	20 (1.6)
Pharmacist	14 (1.1)
Health office	8 (0.7)
Health insurance company	12 (0.1)
