# Supplementary material for: Determinants of tetanus, pneumococcal and influenza vaccination in the elderly: a representative cross-sectional study on knowledge, attitude and practice (KAP)
Source: BMC Public Health. 2016 Feb 4;16:121. doi: 10.1186/s12889-016-2784-8 (PMC4743086; doi:10.1186/s12889-016-2784-8)
Supplement: Supplementary file 4 — Details on results of factor analyses. Description: describes in detail the generated factors which are the basis for the scores used in further analyses including KMO, Bartlett, Eigenvalue, factor loading, included variables, Cronbach’s alpha, mean and standard deviation. (PDF 441 kb) [file 12889_2016_2784_MOESM4_ESM.pdf]

Additional file 4- Details on results of factor analyses

**Knowledge: Promax: KMO=0.6=mediocre; Bartlett<0.001**					
Factor Eigenvalue (% of variance)	**1-Influenza 2.2 (11.3%)**	**2-Diphtheria 1.7 (8.8%)**			
Variable (rotated factor loading)	Recommendation Influenza-vaccination, spontaneous (0.9)	Recommendation Diphtheria-vaccination, spontaneous (0.8)			
	Recommended Influenza-vaccination-time interval (0.9)	Recommended Diphtheria-vaccination-time interval (0.7)			
Cronbachs alpha >= 60 yrs (all)	**0.97 (0.97)**	**0.85 (0.92)**			
Obs /Mean (SD)	**1223 /0.47 (0.83)**	**1223/ 0.24 (0.60)**			
**Attitude towards vaccination: Promax: KMO<0.9=meritorious; Bartlett<0.001**					
Factor Eigenvalue (% of variance)	**1-Interest in further information 7.1 (10.3%)**	**2- Importance 4.3 (6.3%)**	**3-Source of information 2.6 (3.8%)**	**4-Influenza-vaccination+ all vaccination 2.4 (3.5%)**	**5-Barriers 1.8 (2.6%)**
Variable (rotated factor loading)	Interest in further information (1.0)	Measles vaccination important (0.8)	Brochure appropriate (0.6)	Influenza vaccination important (0.7)	Barrier side effects (0.8)
	Interest in further information about period of effect (1.0)	Mumps vaccination important (0.7)	Federal and national state authorities (0.5)	Barrier influenza: no protection (-0.6)	Barrier no protection (0.7)
	Interest in further information about side effects (1.0)	Rubella vaccination important (0.7)	TV appropriate (0.5)	Barrier influenza: disease harmless (-0.5)	Barrier critical reports in media (0.6)
	Interest in further information about mode of functioning (0.9)	Diphtheria vaccination important (0.7)	Newspaper/magazine appropriate (0.5)	Attitude towards vaccination (0.5)	Barrier disease harmless (0.6)
	Interest in further information about recommendations (0.9)	Varicella vaccination important (0.7)	Health office appropriate (0.5)	Barrier influenza: no target group (-0.5)	
	Interest in further information about assumption of costs (0.9)	Polio vaccination important (0.7)	Health insurance company appropriate (0.5)	Barrier influenza: side effects (-0.5)	
	Interest in further information about new developments (0.9)	Pertussis vaccination important (0.6)	Family appropriate (0.5)		
		Meningococci vaccination important (0.4)	Friends appropriate (0.5)		
		Hepatitis B-vaccination important (0.4)	Physician assistance appropriate (0.4)		
Cronbachs	**0.98 (0.97)**	**0.86 (0.83)**	**0.74 (0.68)**	**0.72 (0.60)**	**0.77 (0.77)**

alpha >= 60 yrs (all)					
Obs /Mean (SD)	**1223/ 1.15 (2.43)**	**1223 / 5.51 (2.94)**	**1157 / 5.40 (2.49)**	**1177 / 2.33 (1.81)**	**1210/ 0.28 (0.79)**
**Vaccination related practices; Promax: KMO<0.8=middling; Bartlett<0.001**					
Factor Eigenvalue (% of variance)	**1-Influenza+ any vaccination 4.5 (16.5%)**	**2-Other vaccinations 2.4 (8.8%)**	**3-Vaccination consultation 1.8 (6.6%)**	**4-Vaccination record 1.4 (5.0%)**	**5-Consulted webpage „Impfen-Info.de" 1.3 (4.9%)**
Variable (rotated factor loading)	Influenza vaccination during previous five years (1.0)	Measles vaccination during previous five years (0.8)	Vaccination consultation (1.0)	Knowing where vaccination record is (0.9)	How often consulted webpage „Impfen-Info.de" (0.9)
	Influenza vaccination annually (0.9)	Rubella vaccination during previous five years (0.7)	Vaccination consultation by physician (1.0)	Possession vaccination record (0.8)	Consulted webpage „Impfen-Info.de" (0.8)
	Influenza vaccination ever (0.7)	Mumps vaccination during previous five years (0.7)			
	Any vaccination during previous five years (0.7)	Varicella vaccination during previous five years (0.6)			
	Tetanus vaccination during previous five years (0.4)	Pertussis vaccination during previous five years (0.5)			
		Polio vaccination during previous five years (0.4)			
		Diphtheria vaccination during previous five years (0.4)			
Cronbachs alpha >= 60 yrs (all)	**0.86 (0.81)**	**0.71 (0.80)**	**0.99 (0.98)**	**0.72 (0.62)**	**0.71 (0.78)**
Obs /Mean (SD)	**1206 /2.96 (1.93)**	**1200/ 0.39 (0.88)**	**1220 / 0.44 (0.83)**	**1213 / 2.41 (1.08)**	**1223 / 0.05 (0.42)**
