# Supplementary material for: Determinants of tetanus, pneumococcal and influenza vaccination in the elderly: a representative cross-sectional study on knowledge, attitude and practice (KAP)
Source: BMC Public Health. 2016 Feb 4;16:121. doi: 10.1186/s12889-016-2784-8 (PMC4743086; doi:10.1186/s12889-016-2784-8)
Supplement: Supplementary file 5 — Characteristics of scores. Description: shows the characteristics of the generated scores in terms of mean, standard deviation, minimum, maximum, median and numbers and percentages regarding the dichotomized scores. (PDF 169 kb) [file 12889_2016_2784_MOESM5_ESM.pdf]

Additional file 5- Characteristics of scores

Score	Knowledge	Attitude	Practice
**Obs**	1223	1107	1174
**Mean (SD)**	0.7 (1.0)	14.3 (6.0)	6.2 (3.2)
**Min/Max**	0/4	-4/29	0/23
**Median**	0	14	6.5

Scores, dichotomous

	Knowledge=1	Attitude=1	Practice=1
**N (%)**	441 (36.1)	547 (49.4)	636 (52.0)
